# Adenosine deaminase 2 (ADA2) deficiency masquerading as polyarteritis nodosa

**DOI:** 10.1016/j.jdcr.2025.09.044

**Published:** 2025-10-14

**Authors:** Chelsea Reynolds, Lara Wine Lee, Jodi Dingle, Natasha Ruth

**Affiliations:** a Department of Pediatrics, Medical University of South Carolina, Charleston, South Carolina; b Department of Pediatrics, Prisma Health Children’s Hospital – Midlands, University of South Carolina School of Medicine, Columbia, South Carolina

**Keywords:** aneurysm, deficiency, hemorrhage, pediatrics, polyarteritis nodosa, vasculitis

## Clinical images

### Case description

**Fig 1:**
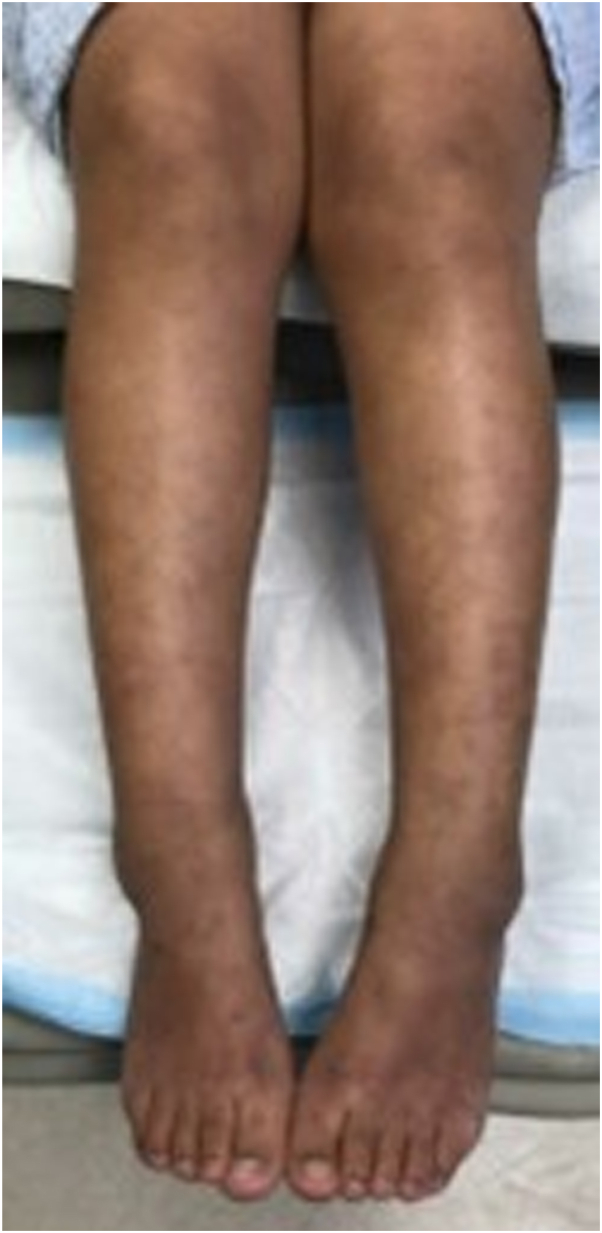
Livedo reticularis and edema of the lower extremities.

An 8-year-old female presented to clinic with a 2-month history of fever, lower extremity edema, and livedo reticularis. Initial lab work was notable for an elevated sedimentation rate at 32. She returned to clinic at 15 years old and had continuation of livedo reticularis, and lower extremity edema (Fig 1). Lab work indicated a sedimentation rate of 24, antinuclear antibody was positive at 1:80 with a negative extractable nuclear antigen panel, and anti-neutrophil cytoplasmic antibodies was negative. Punch biopsy of the livedoid rash showed increased dermal mucin and subcutaneous lymphocytic perivascular inflammation without frank vasculitis. Deeper biopsy revealed neutrophilic vasculitis involving an artery in the subcutaneous fat with surrounding lobular necrosis, consistent with polyarteritis nodosa. She started on prednisone and azathioprine. The following year, she endorsed muscle fatigue with falls, and an MRI of the cervical, thoracic, and lumbar spine were unremarkable, and these symptoms subsequently resolved.

At age 17, she presented with a severe headache and CT angiogram demonstrated a perimesencephalic subarachnoid hemorrhage. A digital subtraction angiography revealed a partially thrombosed basilar perforator aneurysm. There was no clear etiology for the hemorrhage, but it felt likely related to polyarteritis nodosa (PAN), so azathioprine was discontinued, and she started on monthly intravenous cyclophosphamide, then maintained on mycophenolate mofetil. Three months later, she was seen at an outside emergency department with complaints of severe headache. CT showed subarachnoid hemorrhage in the posterior fossa above and below the level of the foramen magnum extending into the outlet foramina. Findings were suspicious for a ruptured vertebral artery and posterior inferior cerebellar artery aneurysm. CT angiogram showed no obvious intracranial aneurysm. She was admitted to the neurosurgical intensive care unit and cerebral angiogram was negative. MRI of her cervical, thoracic, and lumbar spine showed no spinal arteriovenous malformation. A pulse dose of solumedrol was given for presumed poorly controlled PAN. Due to the severity of her central nervous system involvement, which is atypical in PAN, plasma Adenosine deaminase 2 (ADA2) activity levels were sent which were 0.0 mU per mL. Genetic testing demonstrated 2 pathogenic variants in ADA2 (c.139G>C(p.Gly47Arg)) (homozygous). She started on adalimumab and has had complete resolution of symptoms.

**Question: Which of the following clinical features or findings is most suggestive of ADA2 deficiency over PAN?**

**A.** Presence of livedo reticularis and subcutaneous nodules
**B.** Onset of disease in middle childhood with fever and fatigue
**C.** Histological evidence of medium vessel vasculitis on skin biopsy
**D.** Central nervous system involvement, with features like ischemic stroke
**E.** Skin ulcerations with necrosis and leukocytoclastic vasculitis

## Discussion

ADA2 deficiency is an autosomal recessive autoinflammatory disorder that has a spectrum of manifestations. ADA2 deficiency is associated with monocyte-macrophage polarization towards the M1 subset, which is known to promote inflammation and tissue damage.^1^ Inflammatory features such as intermittent fever, rash, and musculoskeletal involvement are common.^2^ Rash may present as livedo racemosa/reticularis, subcutaneous nodules, and erythema multiforme-like lesions.^2^ Skin biopsies can show medium vessel vasculitis as seen in PAN or leukocytoclastic vasculitis.^3^ Inflammation of blood vessels most characteristically involves the central nervous system and neurologic involvement is estimated to occur in 50% to 77% of patients with ADA2.^4^ Central nervous system involvement is rare in PAN and if seen ADA2 deficiency should be considered. PAN is a rare condition in adults and is even less common in children, therefore characterization of pediatric PAN is limited. Children with PAN typically present in middle childhood with fever, fatigue, and various skin findings, including livedo reticularis, subcutaneous nodules, and even skin ulcerations with necrosis.^4^

When ADA2 deficiency is suspected, laboratory confirmation with molecular testing should be undertaken and would indicate low (<5% of normal) or undetectable ADA2 catalytic activity.^5^ Treatment of ADA2 deficiency involves anti-tumor necrosis factor alpha agents, which prevent and eliminate manifestations of autoinflammatory disease, reduce the risk of ischemic stroke, and relieve immunodeficiency.^5^

## Conflicts of interest

None disclosed.
